# Pain Induced during Both the Acquisition and Retention Phases of Locomotor Adaptation Does Not Interfere with Improvements in Motor Performance

**DOI:** 10.1155/2016/8539096

**Published:** 2016-12-08

**Authors:** Jason Bouffard, Laurent J. Bouyer, Jean-Sébastien Roy, Catherine Mercier

**Affiliations:** ^1^Department of Rehabilitation, Université Laval, 1050 Avenue de la Médecine, Québec, QC, Canada G1V 0A6; ^2^Centre Interdisciplinaire de Recherche en Réadaptation et Intégration Sociale (CIRRIS), 525 Boul. Hamel, Québec, QC, Canada G1M 2S8

## Abstract

Cutaneous pain experienced during locomotor training was previously reported to interfere with retention assessed in pain-free conditions. To determine whether this interference reflects consolidation deficits or a difficulty to transfer motor skills acquired in the presence of pain to a pain-free context, this study evaluated the effect of pain induced during both the acquisition and retention phases of locomotor learning. Healthy participants performed a locomotor adaptation task (robotized orthosis perturbing ankle movements during swing) on two consecutive days. Capsaicin cream was applied around participants' ankle on both days for the Pain group, while the Control group was always pain-free. Changes in movement errors caused by the perturbation were measured to assess global motor performance; temporal distribution of errors and electromyographic activity were used to characterize motor strategies. Pain did not interfere with global performance during the acquisition or the retention phases but was associated with a shift in movement error center of gravity to later in the swing phase, suggesting a reduction in anticipatory strategy. Therefore, previously reported retention deficits could be explained by contextual changes between acquisition and retention tests. This difficulty in transferring skills from one context to another could be due to pain-related changes in motor strategy.

## 1. Introduction

Pain can influence the way we move in several manners, ranging from total avoidance of potentially harmful movements to more subtle changes in muscle recruitment [1]. While several studies have described the immediate effect of pain on motor performance [2], its effect on motor learning has been less investigated [3–10]. Among the studies who did look at the effect of pain on motor learning, only a few have considered its impact on the retention of new motor skills [8–10], rather than simply looking at improvement during practice (i.e., skill acquisition).

The impact of pain on locomotor learning is of particular clinical importance, given that neuropathic pain is highly prevalent in populations that have to perform locomotor learning as part of their rehabilitation, such as patients with incomplete spinal cord injury or lower limb amputees starting to use a prosthesis [11–13]. The only study so far that has looked at the effect of pain on a locomotor learning task showed that cutaneous pain induced by topical application of capsaicin (an experimental model of neuropathic pain) impairs the retention of motor learning despite normal performance during the acquisition phase [8]. In this study, pain was applied only during initial training (motor acquisition) and subjects were pain-free when retested for retention on the following day [8]. Based on these results, it has been suggested that cutaneous pain could interfere with neural processes associated with consolidation of motor learning.

An alternative hypothesis however is that as pain alters the context in which motor training occurs, being tested in the same task but in the absence of pain might in some sense be considered as a transfer test rather than a retention test. Therefore, poor retention might potentially be explained by changes in the pain context between motor acquisition and retention testing rather than by an interference with the consolidation process per se. According to the specificity of practice hypothesis [14], it is expected that the performance of participants in a retention test will be optimized if the conditions of testing are identical to the conditions of skill acquisition. In the central nervous system (CNS), sensory information available during motor practice would be associated with the goal of the task and the state of the motor system to form a representation of the motor skill, which would contribute to the specificity of practice effect [14, 15]. Another aspect that might impact the ability to transfer a motor skill from a “pain context” to a “pain-free context” is the fact that pain has been reported to influence motor strategies used during motor adaptation tasks, even when the global performance itself is not affected [9].

The objective of the present study was to evaluate the effect of tonic experimental pain on performance and motor strategies used during the acquisition and retention phases of motor learning in a locomotor adaptation task. In contrast to our previous study [8], pain in the current was induced during both phases of motor learning. If pain directly interferes with processes involved in the consolidation of motor skills, impaired retention with pain should be observed as previously [8]. Alternatively, the absence of impaired retention would support processes involved in the specificity of practice hypothesis described above.

## 2. Method

Thirty-nine healthy participants were recruited among the university student population. Participants included in the study did not report any pain unrelated to the experimental pain stimulus and were able to achieve stable gait with the robotized orthosis. Eligible participants were randomly allocated to a Pain and a Control group, performing the motor task with or without experimental pain, respectively. However, the Control group was voluntarily oversampled, and it is used as the comparison group for several studies. Technical problems delayed the experiment for two participants of the Pain group, which resulted in their pain vanishing before the adaptation phase. They were therefore excluded from the analyses. The final sample was composed of 24 participants in the Control group (10 women, 25.8 ± 0.85 years old) and 13 in the Pain group (8 women, 26.1 ± 1.15 years old). Groups did not differ in terms of age (*t*-test: *p* = 0.857) or sex (Khi-2: *p* = 0.248). All participants provided their written informed consent and the ethics institutional review board approved the project (Institut de Réadaptation en Déficience Physique de Québec, Project #2010-212).

### 2.1. Experimental Procedure

Participants performed the same locomotor adaptation task on two consecutive days. Motor acquisition was evaluated on Day 1 and retention on Day 2. The locomotor adaptation task consisted in walking on a treadmill while overcoming a perturbation of the ankle movement applied by a robotized ankle-foot orthosis (rAFO) [16, 17]. In such task, the perturbation initially causes large deviations in ankle trajectory, termed movement errors. When continuously exposed to the perturbation, participants adapt to the perturbation by modifying their motor behaviour and gradually reduce their movement error through the training session. When the same task is performed after a delay without training, participants' performance is usually better than their performance on their first exposure to the perturbation, demonstrating retention of motor learning [8, 18].

**Figure 1:**
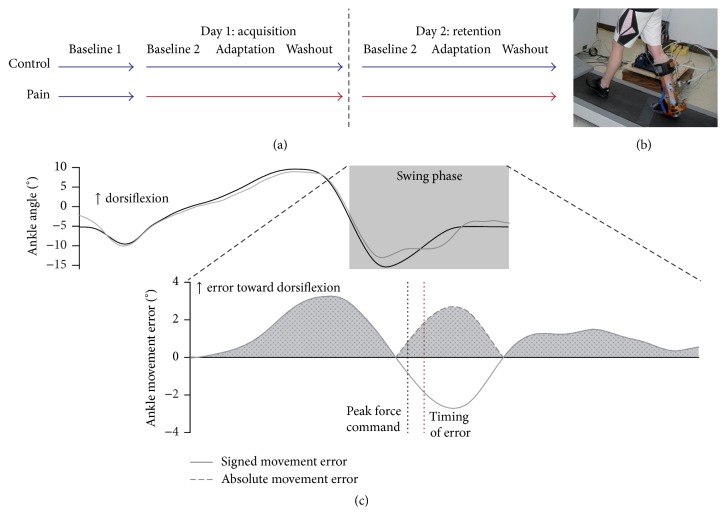
(a) General protocol of the experiment: blue and red arrows illustrate phases of the experiment performed without and with pain, respectively. (b) Robotized ankle-foot orthosis (rAFO). (c) Ankle kinematics outcome measures: the upper panel presents ankle angle during baseline gait (black) and adaptation (gray) for the whole stride duration. The lower panel illustrates the signed and absolute ankle movement error during the swing phase. The gray shaded area illustrates the mean absolute error outcome measure. The black and red dotted lines show the peak force command and the timing of movement error, respectively.

During all experimental procedures (Figure 1), participants walked on a treadmill at a speed of 1 m/s while wearing the rAFO on their right lower limb. On Day 1, all participants began the experiment by walking normally (rAFO actively cancelling its own inertia in order to allow natural gait [16]) for 5 to 10 minutes without any painful stimulation (Baseline 1). This allowed the quantification of participants' normal gait pattern with the rAFO when they are free of pain. Afterward, the main experiment consisted of 15 to 20 minutes of treadmill walking without interruption. Pain was induced just before this walking period for the Pain group. During the first 5 to 10 minutes of the main experiment, participants walked normally as in Baseline 1 (Day 1: Baseline 2; Day 2: baseline). Then, the rAFO applied a force field resisting right ankle dorsiflexion during midswing (parabolic force field, peak amplitude of 4.8 ± 0.1 Nm at 81 ± 1% of gait cycle, 150 ms duration) at each stride for 5 minutes (adaptation) [8, 19]. Participants were not told about the exact time at which the force field would be turned on. They were instructed to “overcome the perturbation in order to walk as normally as possible.” Finally, participants walked again without the force field during 5 minutes in order to recover their normal walking pattern before leaving the laboratory (washout).

### 2.2. Experimental Pain Induction

A ~1 cm wide band of capsaicin cream (1%) (~1 mm thick) was applied around the right ankle of Pain group's participants on both days, between Baseline 1 and Baseline 2 on Day 1 and before baseline on Day 2. Participants were asked to rate the intensity of their pain verbally on a Numerical Rating Scale from 0 (no pain) to 10 (worst pain imaginable) every 3 minutes throughout the experiment. The main experiment started once pain intensity reached a plateau (~30 minutes). On Day 1, a 30-minute wait period was imposed to the Control group between Baseline 1 and Baseline 2 for intergroup consistency.

### 2.3. Data Collection

Relative ankle angle in the sagittal plane was recorded with an optical encoder attached to the rAFO. A load cell placed in series with the rAFO's actuator recorded the forces applied to subjects' ankle. A custom-made pressure sensor placed under the right heel served as a footswitch. Bipolar surface electromyographic (EMG) activity was recorded from right tibialis anterior (TA; ankle dorsiflexor) and soleus (SOL; ankle plantarflexor) muscles. The electrodes were placed on shaved and cleaned skin in the location recommended by SENIAM for the SOL muscle [20]. For the TA, the electrodes were placed just under the calf band of the rAFO, as close as possible to the muscle belly. Electrode placement was marked on participants' skin on Day 1 to ensure between days consistency in EMG measurement. EMG signals were amplified 2000 times (custom amplifier; 10–500 Hz Bessel filter) and all channels were sampled and stored on a desktop computer at 1 kHz/channel using custom data acquisition software.

### 2.4. Data Analysis

EMG data were digitally filtered using a 2nd-order zero-lag Butterworth filter (20–450 Hz bandwidth) and rectified. Thereafter, an envelope was extracted using a 9-point moving average filter [21]. Ankle angle data were filtered with a 2nd-order zero-lag 15 Hz low-pass Butterworth filter. Relative ankle angle was analysed in a period slightly longer than the swing phase: data were synchronised from the middle of the push-off to the right heel strike and time was normalised on 1000 points [8]. EMG analysis window was extended by 30% to include the beginning of TA stance-to-swing burst's onset.

#### 2.4.1. Ankle Kinematics Outcome Measures

A baseline ankle angle template was constructed for each participant by averaging point-by-point ankle angle data for 45 of the last 50 strides of the baseline period (the five less representative strides were removed to limit outlier influence). On Day 1, Baseline 2 data were used to generate baseline ankle angle template. Then, ankle movement error curves were computed by subtracting the baseline template from each stride of the adaptation period. Note that ankle movement error curves of a given day were generated using baseline template of the same day.

Two different variables were derived from these error curves: (1) the mean absolute error, reflecting the general performance of the subject (i.e., the ability to walk “as normally as possible”); and (2) the relative timing of error (providing insights on motor strategies).

The mean absolute error was calculated for each stride of the adaptation period by averaging the rectified movement error curve during the whole swing phase [8].

The relative timing of error, a measure of the temporal center of error distribution relative to the peak force command, was calculated using the following equation for each stride of the adaptation:

(1) Relative timing of error=∑i=11000Errori×i∑i=11000Errori−Peak force command,

where Error_*i*_ is the absolute amplitude at the *i*th data point of the movement error curve and Peak force command is the data point when the force command reached its peak value. This variable provides information about the strategy used to overcome the perturbation during adaptation. Smaller relative timing of error suggests that participants used a more anticipatory strategy (i.e., movement is mainly modified* in preparation for* the perturbation) while larger relative timing of error suggests that participants are more reactive (i.e., movement is mainly modified* in response to* the perturbation).

#### 2.4.2. Electromyography Outcome Measures

**Figure 2:**
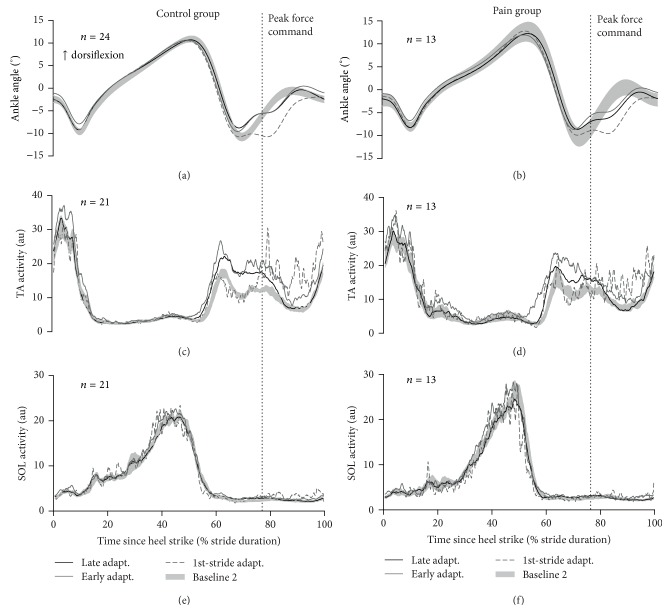
Summary of the results of the acquisition of motor skill (Day 1): (a), (c), and (e) show Control group averaged ankle angle (a), TA activity (c), and SOL activity (e) signals. (b), (d), and (f) present the same signals for the Pain group. The vertical dotted lines indicate the timing of the peak force command during the adaptation period. The shaded gray areas present each group baseline data (mean ± SEM). The gray lines illustrate the first stride (dashed line) and strides 2 to 11 (early adaptation; full line) of the adaptation period while the black line presents data during late adaptation (strides 151 to 200).

Visual inspection of EMG data (Figures 2(c)–2(f)) revealed that changes in EMG activity during the adaptation period were limited to the TA muscle as observed in Blanchette et al. 2011 [19]. Therefore, EMG analyses only focused on this muscle. Changes in TA activity during adaptation were quantified by computing the TA ratio during this period relative to baseline. A TA ratio vector was calculated using a point-by-point ratio of TA activity during the adaptation period over its activity during baseline. TA ratio was summarised in three outcome measures by averaging the TA ratio vector for the whole analysis window duration, as well as before and after the peak force command. The former variable informs about the global changes in EMG during the adaptation. The latter two variables are related to anticipatory and reactive strategies used by participants to overcome the force field, respectively. TA ratios were linearized with a log_2_ transformation for statistical analyses while descriptive statistics on untransformed data are presented in the text.

### 2.5. Statistics

To quantify the effect of pain on the acquisition and retention of motor learning, 3-way repeated measure ANOVAs (time: early (strides 2 to 11) versus late adaptation (strides 151 to 200), day: Day 1 versus Day 2, group: Control versus Pain) were used for the mean absolute error and TA ratios. The first stride of the adaptation period was not a priori included in the statistical analysis, as participants did not know when the perturbation would be turned on. A generalised estimation equation for gamma distributions with log links was applied to the relative timing of error variable using the same design (time × day × group) [22]. The Benjamini-Hochberg correction for multiple comparisons was applied for post hoc analyses [23]. Data are presented in the text and figures as mean ± standard error of the mean (SEM). Level of significance was set at *p* < 0.05.

## 3. Results

### 3.1. Experimental Pain Intensity

The intensity of the pain induced by capsaicin was consistent between days (Day 1: 5.6 ± 0.7; Day 2: 5.5 ± 0.7; ICC: 0.842; paired *t*-test *p* = 0.787* *), confirming that the Pain group participants were in similar conditions for the evaluation of motor acquisition and retention.

### 3.2. Effect of Pain on Baseline Gait Parameters

Consistent with previous report [8], the mean absolute ankle angle difference and TA activity ratio between Baseline 1 (measured pain-free in both groups) and Baseline 2 (assessed with pain in the Pain group) were not different between groups (ankle kinematics: *t*-test *p* = 0.147, TA activity: *t*-test: *p* = 0.916). These results indicate that any difference in the acquisition of the motor adaptation is unlikely to be accounted for by a direct impact of pain on baseline gait.

### 3.3. Effect of Pain on Motor Learning

Figure 2 qualitatively illustrates the results on the effect of pain during the acquisition phase of motor learning (i.e., Day 1). Quantitative analyses are presented in the subsequent sections. The upper panels (Figures 2(a) and 2(b)), depicting ankle kinematics, shows that participants in both groups initially had large movement errors on their first stride of exposure to the perturbation but then quickly modified their motor behaviour. Actually, most kinematic changes occur in the first strides of exposure, as there is a larger difference between the first stride and early adaptation (average of strides 2–11) than that between early and late adaptation (average of strides 151–200). The middle panels (Figures 2(c) and 2(d)) show that this reduction of movement errors is achieved through an increase in TA activity. On the first stride of the adaptation period, increase in TA activity occurs only late in the swing phase (after the vertical line depicting the peak force command). This is explained by the fact that the perturbation is unexpected, and as a result the response to the perturbation is purely reactive. On the following strides (i.e., during early adaptation), the EMG increase is observed earlier in the swing phase, reflecting a more anticipatory strategy. The large increase in TA activity initially observed slightly diminishes over time (i.e., from early (strides 2–11) to late (strides 151–200) adaptation), reflecting fine-tuning of the motor behaviour through practice. The lower panel of this figure (Figures 2(e) and 2(f)) shows that no significant changes in SOL muscle activity occurred during the adaptation period.

#### 3.3.1. Ankle Kinematics

**Figure 3:**
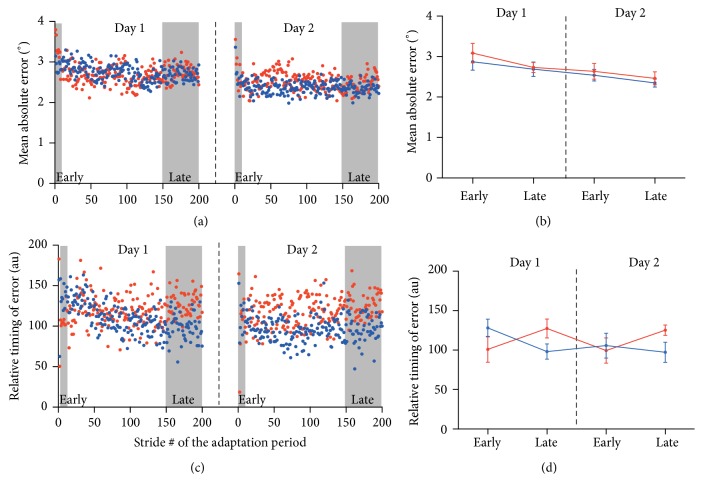
Results for ankle kinematic outcome measures: (a) and (c) present Control (blue) and Pain (red) group averaged time course of the mean absolute error (a) and relative timing of error outcome measures (c) for each day. Gray areas highlight the strides used for the computation of early and late adaptation presented in (b) and (d) (mean ± SEM).

Results for the mean absolute error are presented in Figure 3(a) depicting the stride-by-stride time course during adaptation and Figure 3(b) showing average value for this variable during early and late adaptation on each day. A day effect was observed, showing an improvement in performance for both groups from Day 1 to Day 2 (effect of day: *p* < 0.001). However, there was no time, group, or interaction effect for the mean absolute error (all *p* > 0.156). It is important to note that the absence of effect of time is related to the fast improvement of participants' performance in opposition to an absence of improvement. Indeed, if the early adaptation epoch is replaced by the first adaptation stride in the ANOVA, the effect of time becomes significant showing the clear improvement in performance during the adaptation period (*p* < 0.001).

The time course of relative timing of error variable and the average for early and late adaptation epochs are presented in Figures 3(c) and 3(d), respectively. Results show a significant time × group interaction (*p* = 0.005; all other *p* > 0.233). Post hoc analyses were therefore performed by averaging Day 1 and Day 2 data and are presented in Table 1. For the Control group, the relative timing of error changes toward smaller values from early to late adaptation suggesting that participants without pain used a more anticipatory strategy to overcome the perturbation with practice. In contrast, for participants with pain, the relative timing of error tended to change toward larger values during the adaptation period suggesting less anticipatory strategy with practice. Moreover, the Pain group tended to use less anticipatory strategy during late adaptation then the Control group.

#### 3.3.2. EMG

Significant effects of time were observed for all three TA outcome measures (ANOVA time: *p* ≤ 0.001). No effects of group, day, or interactions were detected (all *p* > 0.134). Therefore, the effect of time is presented with more detail for the whole sample averaged across days and only for the global TA ratio variable. After a rapid and important increase in TA activity during early adaptation (Early adaptation = 138 ± 6% baseline TA EMG), participants slightly decreased their TA activity (Late adaptation = 113 ± 3% baseline TA EMG). Importantly, however, TA EMG activity remained higher during the adaptation period compared to baseline at all time points (one sample *t*-test: total TA ratio versus 0, all *p* < 0.006).

## 4. Discussion

Results of the present study show that cutaneous pain does not interfere with global performance during the acquisition and retention of a locomotor adaptation when skill acquisition and retention tests are both performed in the same context (i.e., with pain). Although global performance was unaffected by pain, the pattern of kinematic errors (relative timing of error) suggests that participants in the Pain group used a different motor strategy in order to overcome the force field compared to those of the Control group. Participants in the Control group shifted the relative timing of error with practice toward lower values suggesting a greater usage of anticipatory strategies (or a greater decrease in reactive errors) while the Pain group tended to show the opposite behaviour. However, no between groups differences were observed at any time point of the experiment on this variable despite the presence of a significant time × group interaction. Moreover, changes in TA activity across time were not different with or without pain. It is therefore difficult to interpret the effect of cutaneous pain on motor strategies used during locomotor adaptation thoroughly. The relatively small sample size of the study may have limited the power for some comparisons (especially for relative timing of error post hoc where trends were observed).

The fact that cutaneous pain did not influence the global performance of participants during the* acquisition* of the locomotor adaptation task is in line with our previous findings [8]. However, contrary to the results obtained with capsaicin applied only during the acquisition of motor skills [8], no interference with the retention of motor learning was observed when both the acquisition and retention were tested with pain. Those opposite results between these two studies using otherwise similar experimental paradigms suggest that the motor skills acquired while training in the locomotor adaptation task can be consolidated into the CNS even in the presence of pain. However, based on the results of Bouffard et al., 2014, the addition of cutaneous pain during motor acquisition appears to modify the representation of the motor skills into the CNS sufficiently to induce retention (or transfer) deficit when the same task is performed again without pain. The fact that participants of each group adopted different motor strategies during the locomotor adaptation emphasises the fact that motor skills can be acquired differently with pain although the task goal may still be reached. Previous studies have shown reorganisation of muscle activity with pain or alterations in kinematics while maintaining the global motor performance intact in line with changes in motor strategy observed in the present study [9, 24, 25].

The performance decrement with the change in context between the acquisition and retention of a motor skill is in accordance with the specificity of practice hypothesis [14]. In the force field adaptation literature, a concept similar to the specificity of practice is often studied by evaluating the effect of matching various contextual cues to different pattern of force fields that are normally impossible to learn simultaneously because they require opposite motor strategies (e.g., clockwise versus counterclockwise perturbation during reaching movements). Reduced interference when each force field is associated with a different contextual cue is interpreted as a proof that the contextual cues allow the independent coding of motor skills involved for each adaptation [15, 26, 27]. Different contextual conditions can be manipulated to affect performance during retention tests and can be analyses in relation to the specificity of practice hypothesis. Those contextual conditions can be grouped into three categories: task-related sensory manipulations, task-related motor state manipulations, and task-unrelated (or indirectly related) manipulations. The impact of pain on factors involved in each of these categories will be addressed in order to give potential explanation of the present results.

Removing, adding, or disrupting the primary sources of feedback between the acquisition of a motor skill and retention test leads to results in line with the specificity of practice hypothesis [15, 28, 29]. It has been hypothesised that, during the acquisition of a motor skill, individuals learn to combine all feedback sources available to guide their performance. By removing (or adding) a dominating source of feedback during a transfer test, participants need to modify the way they integrate the information on which they rely during the motor task, leading to a performance decrement [14]. Disrupting a source of feedback without completely removing it also leads to similar results [15, 30]. In the present study, it is unlikely that participants learnt to rely on the sensory feedback caused by capsaicin application as the pain induced was tonic and unrelated to movements. However, pain can impair proprioceptive and cutaneous perception [31, 32], which are the primary sources of feedback during the locomotor adaptation task studied.

Other authors suggested that, during motor learning, the motor commands needed to reach the task goal are mapped as a function of the limb state [33]. Modifying the limb state during motor training, for instance, by modifying the location of reaching movement during motor adaptation, results in findings consistent with the specificity of practice hypothesis [15, 27]. In line with this view, it could also be expected that a manipulation of the input-output properties of the neural structures involved in the control of movement would influence the way motor skills are coded. Pain can exert such influence as some studies have shown changes in the excitability of different spinal and cortical neural network involved in motor control [34–36]. If an individual trains to a motor task with pain, he would learn to associate its motor commands to given outcomes. If pain disrupts the excitability of the neuronal structures involved in the motor task, this input-output mapping would be equally disrupted. Thus, when the individual would be tested for retention without pain to the same motor task, he would recover an erroneous control function, which would result in a performance decrement.

The effect of pain on variables directly involved in the motor task such as the feedback sources or the state of the motor system is not the only explanation for its context dependent effect on retention of locomotor adaptation. Manipulation of contextual characteristics not directly impacting the motor task can influence retention/transfer testing as well [15, 26, 37, 38]. For instance, it was shown that participants trained in various novel motor tasks (golf putting, wall climbing, or basketball free throw) in a low anxiety generating environment performed worse when tested in a high anxiety generating environment than participants who trained with anxiety. Conversely, participants who trained with anxiety had lower performance when subsequently tested without anxiety [37, 38]. This suggests that the variability between contexts where a skill is trained versus retested (or used in everyday life) might have a more deleterious effect on retention (or transfer) than a* negative* context in itself (e.g., pain, anxiety). This potentially has significant clinical implications given that, in several populations with pain, pain intensity (as well as mood) is often quite variable from one day to another [39–41].

## 5. Conclusion

While cutaneous pain during locomotor training was previously reported to interfere with retention when assessed in pain-free conditions, the results of the present study show that it does not prevent next-day retention when the pain context is similar between days. Together, these results suggest that the retention deficit previously reported could be explained by changes in contextual conditions between motor acquisition (with pain) and retention test (without pain), rather than to a direct impact of pain on the consolidation of motor skills. The fact that motor strategies used to improve performance appear to be modified by pain might contribute to the difficulty to transfer the new skills from one context to another. This has clinical implications given that pain intensity is known to be variable over time in several populations undergoing physical rehabilitation [40, 41].

## Figures and Tables

**Table 1:** Post hoc analyses for the time × group interaction on the relative timing of error variable. Uncorrected *p* values are presented. According to the Benjamini-Hochberg procedure, the critical *p* value = *α*/*m∗i*, where m corresponds to the number of hypotheses tested (*m* = 4) and *i* corresponds to the rank of the tested hypothesis based on the uncorrected *p* value.

	Between groups		Within group
	Control versus Pain		Early versus late
Early adaptation	*p* = 0.321	Control	*p* = 0.007^a^
Late adaptation	*p* = 0.076	Pain	*p* = 0.048^b^

^a^Critical *p* value = 0.05/4*∗*1 = 0.0125; ^b^Critical *p* value = 0.05/4*∗*2 = 0.025.
